# Heart Rate Variability from Wearable Photoplethysmography Systems: Implications in Sleep Studies at High Altitude

**DOI:** 10.3390/s22082891

**Published:** 2022-04-09

**Authors:** Paolo Castiglioni, Paolo Meriggi, Marco Di Rienzo, Carolina Lombardi, Gianfranco Parati, Andrea Faini

**Affiliations:** 1 IRCCS Fondazione Don Carlo Gnocchi ONLUS, 20148 Milan, Italy; pmeriggi@dongnocchi.it (P.M.); mdirienzo@dongnocchi.it (M.D.R.); 2 Department of Cardiovascular, Neural and Metabolic Sciences, Istituto Auxologico Italiano, IRCCS, 20149 Milan, Italy; c.lombardi@auxologico.it (C.L.); gianfranco.parati@unimib.it (G.P.); a.faini@auxologico.it (A.F.); 3 Department of Medicine and Surgery, University of Milano-Bicocca, 20126 Milan, Italy

**Keywords:** spectral analysis, self-similarity, detrended fluctuation analysis, SampEn, multiscale entropy, sleep, breathing disorders, polysomnography, HRV

## Abstract

The interest in photoplethysmography (PPG) for sleep monitoring is increasing because PPG may allow assessing heart rate variability (HRV), which is particularly important in breathing disorders. Thus, we aimed to evaluate how PPG wearable systems measure HRV during sleep at high altitudes, where hypobaric hypoxia induces respiratory disturbances. We considered PPG and electrocardiographic recordings in 21 volunteers sleeping at 4554 m a.s.l. (as a model of sleep breathing disorder), and five alpine guides sleeping at sea level, 6000 m and 6800 m a.s.l. Power spectra, multiscale entropy, and self-similarity were calculated for PPG tachograms and electrocardiography R–R intervals (RRI). Results demonstrated that wearable PPG devices provide HRV measures even at extremely high altitudes. However, the comparison between PPG tachograms and RRI showed discrepancies in the faster spectral components and at the shorter scales of self-similarity and entropy. Furthermore, the changes in sleep HRV from sea level to extremely high altitudes quantified by RRI and PPG tachograms in the five alpine guides tended to be different at the faster frequencies and shorter scales. Discrepancies may be explained by modulations of pulse wave velocity and should be considered to interpret correctly autonomic alterations during sleep from HRV analysis.

## 1. Introduction

The interest in photoplethysmography (PPG) for monitoring subjects at risk of cardiovascular events during sleep is rapidly increasing. This is due to the easy integration of PPG sensors into wearable systems for collecting clinically valuable signals while interfering as little as possible with sleep. By using lights at wavelengths with different absorptions in oxygenated and deoxygenated hemoglobin, PPG allows measuring the oxygen saturation in the blood, which is useful for detecting sleep-related breathing disorders [1] or an impaired pulmonary function in heart failure [2]. PPG devices can also serve as heart-rate monitors measuring the inter-beat interval between changes in light intensity. In this way, they allow assessing heart rate variability (HRV) to detect autonomic alterations [3,4], which is also particularly important in sleep-related breathing disorders [5] or heart failure [6]. Therefore, the possibility to simultaneously measure HRV and blood oxygen saturation unobtrusively during sleep makes wearable PPG systems promising tools for long-term monitoring of cardiac patients, or for evaluating treatments and rehabilitation programs in these patients.

However, there are limits in using the PPG for HRV analysis. First, pulse-rate modulations depend not only on the modulations of the time interval between depolarizations of the left ventricle but also on modulations of the ejection time and transmission time of the wave from the heart to the measuring site. These variability components are absent in the dynamics of the R–R intervals of the electrocardiogram (ECG), the gold standard in HRV studies. Second, vasomotion may change the shape of the PPG waveform, making it difficult to define precisely the duration of each wave. Third, the PPG signal may be more easily affected than the ECG to movements artifacts and may become undetectable at fingers and toes in case of exaggerated responses to cold or stress. Therefore, different studies evaluated how well PPG-based devices quantify the HRV in comparison to ECG devices: readers may find recent reviews in [7,8,9,10]. Validation studies do not provide univocal results and the HRV quality from PPG systems appears to depend on the experimental set-up [11], the PPG measuring site [12], gender and age [13], and on the pulse wave feature that defines the interval between beats [14,15]. In patients, it may also depend on the nature of the disease [7,11]. In addition, most of the validation studies focused on short-term HRV components, not considering more recent multiscale or broadband approaches to HRV analysis, and employed laboratory devices not practical to be used at home, such as full polysomnography systems during sleep studies.

For these reasons, our work aims to evaluate how PPG-based wearable systems used in real-world environments measure HRV during sleep in conditions where respiratory disturbances or autonomic alterations are expected. For this aim, we considered the physiological human model of sleeping at high altitudes. The hypobaric hypoxia associated with this condition may change the sensitivity of the chemoreceptors regulating the breathing function dramatically and may induce autonomic alterations that reproduce the sleep disorders affecting some patients with cerebrovascular diseases or heart failure [16,17]. In addition, this experimental set-up represents, per se, a useful application of wearable PPG systems. Millions of people live or spend a stay of some days at high altitudes for leisure or work. All of these individuals may be exposed to acute or chronic mountain sickness that in some cases may have severe outcomes during sleep. When symptoms of altitude sickness are present, monitoring the autonomic function and the oxygen saturation during sleep with wearable PPG monitors could be important.

Thus, this real-field study compared HRV measures derived from PPG and ECG signals recorded by a wearable system in participants sleeping at high altitudes. In particular, our study aimed at evaluating the discrepancies between PPG- and ECG-derived measures of HRV in subjects with sleep-related breathing disorders, as induced by hypobaric hypoxia, and whether such discrepancies may impede the correct quantification of the HRV changes that occur at high altitudes during sleep.

## 2. Materials and Methods

### 2.1. Participants and Data Collection 

The study is based on data collected with wearable PPG devices during two scientific expeditions of the HIGHCARE projects (www.highcareprojects.eu accessed on 6 April 2022). The experiments of the expeditions, both conducted in agreement with the principles of the Declaration of Helsinki, allow us to investigate distinct aspects related to the feasibility of HRV analysis by PPG during sleep. The HIGHCARE-ALPS 2010 expedition provides us with data on a relatively large group of participants (*n*= 21, see details below) sojourning at a high altitude on Mt. Rosa, in the Italian Alps. These data allow us to quantify the discrepancies between PPG-derived and ECG-derived measures of sleep HRV in human models that simulate sleep-related breathing disorders with a broad range of severity. By contrast, the HIGHCARE-HIMALAYA expedition provides us with data from a small group of professional mountaineers (*n* = 5) who attempted to climb the Mt. Everest summit. We also considered this small group of participants for two reasons. First, nighttime sleep recordings at such high altitudes are extremely rare and the data in five participants measured twice while sleeping in tents at very high camps provide precious information for understanding the feasibility of sleep studies by wearable PPG devices even in extreme high-mountain environments. Second, although the sample size is too small for obtaining solid statistical conclusions on the effects of high altitude on sleep HRV, it nevertheless may qualitatively describe whether the alterations quantified by PPG and ECG are comparable and consistent with each other. Details on the two expeditions follow.

Mt. Rosa expedition (EudraCT No. 2010-019986-27). In the summer of 2010, 41 volunteers of the Italian Alps expedition ascended in about 28 h from Milan to the Margherita Hut (4554 m a.s.l. on Mt. Rosa, Italian Alps), where they spent five days participating in a randomized study on the efficacy of acetazolamide vs. placebo as a treatment against symptoms of acute mountain sickness [18]. Acetazolamide is expected to modify the chemoreflex response to hypobaric hypoxia. Details on the recruitment, drug and placebo administration, and general characteristics of the whole group of participants are reported in the supplemental document. All participants underwent polysomnography starting on the evening of the third day of their stay. Twenty-one of them (11 males and 10 females, age between 24.4 and 42.7 years, body mass index between 17.2 and 27.1 kg/m^2^, 12 taking acetazolamide and 9 a placebo) were instrumented with a wearable monitoring system called Maglietta Interattiva Computerizzata (MagIC). MagIC included two woven ECG electrodes (sampled at 200 Hz and 12 bit), a textile plethysmograph for measuring respiratory movements of the thorax (50 Hz, 12 bit), and a triaxial accelerometer (50 Hz, 12 bit), and it was connected to a Nonin Xpod^®^ device (Nonin Medical, Inc., Plymouth, MN, USA), which used red and infrared LEDs to record oxygen saturation (3 Hz, 8 bit) and a finger plethysmogram (75 Hz, 8 bit) [19]. The recording lasted about 10 h and covered the whole period of night-time sleep.

Mt. Everest expedition (EudraCT No. 2008-000540-14). Among the participants of the Himalayan expedition, we considered five male alpine guides (age between 29 and 54 years, body mass index between 21.1 and 24.2 kg/m^2^) who attempted to reach the summit of Mt. Everest while being monitored during the night by the MagIC device (details on the recruitment, drug and placebo administration, and general characteristics of the whole group of participants are reported in the supplemental document). None of these five participants was taking any drugs or non-pharmacological interventions against mountain sickness. They performed the first nighttime recording in Milan (at a height of 120 m, approximately at sea level) wearing the MagIC system with the finger PPG device (see Mt. Rosa expedition section). After flight transportation to the village of Namche Bazar and a six-day trek, the five mountaineers reached the Mt. Everest base camp (5400 m) for a 12-day acclimation before moving to Camp 1 at 6000 m a.s.l. Here they performed a second nighttime recording with MagIC, sleeping within a tent. Due to adverse weather conditions, only four of the five mountaineers reached Camp 2 at 6800 m a.s.l., where they performed the third nighttime recording in the tent before returning to the base camp, giving up climbing to the summit. All of the recordings lasted about 10 h and covered the whole nighttime sleep.

### 2.2. Pre-Elaboration

The thoracic movements by the triaxial accelerometer and the textile thoracic band provided information on the respiratory efforts and the PPG identified episodes of oxygen desaturation. These pieces of information were processed together automatically by the Somnologica software (Medcare Flaga, Reykjavik, Iceland) that calculated the apnea-hypopnea index accordingly to the current guidelines [20]. The evaluation of the apnea-hypopnea index with the MagIC device was previously validated vs. a standard cardiorespiratory monitor [17].

To derive the tachograms for HRV analysis, the PPG signal was resampled at 200 Hz after linear interpolation to have the same sampling rate as the ECG. A segment of at least one-hour duration during sleep was visually selected looking for stable periods of the triaxial accelerometers in lying position. Premature beats and artifacts were identified visually on the selected ECG and PPG segments separately and removed. The percentage of removed signal over the total duration of the selected segment was calculated separately for each ECG and PPG signal.

**Figure 1:**
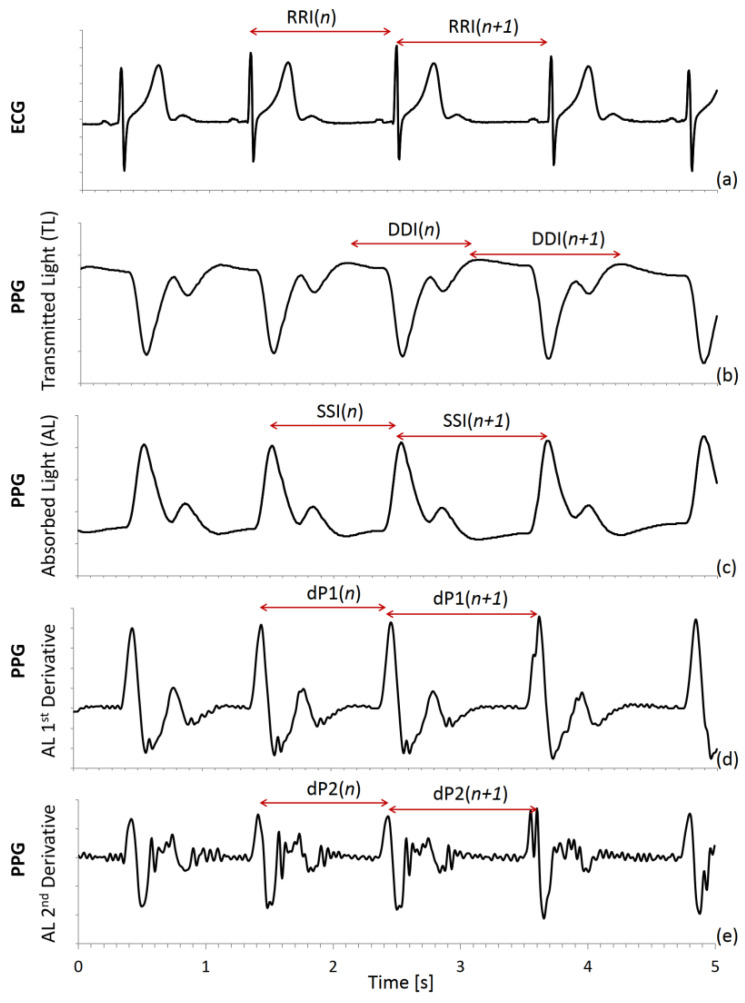
ECG and PPG tachograms. Derivative-and-threshold algorithms are applied to (**a**) ECG, (**b**) intensity of transmitted light (TL) on the finger PPG sensor, (**c**) absorbed light (AL = −TL), (**d**) AL 1st and (**e**) 2nd derivative. RRI is the reference; PPG tachograms are: DDI = interval between TL maxima; SSI = interval between AL maxima; dP1 = interval between maxima of AL 1st derivative; and dP2 = interval between maxima of AL 2nd derivative.

A derivative-and-threshold algorithm was applied to the ECG to identify the R peak, with parabolic interpolation to refine the R wave fiducial point [4], and the R–R intervals (RRI) were calculated beat by beat (Figure 1a). As to the photoplethysmogram, a similar derivative-and-threshold algorithm was applied to the photodetector signal, which measures the intensity of the transmitted light (TL) from the LED through the finger. A beat-to-beat series was extracted as the intervals between the maxima (DDI) in the diastolic phase of the heartbeat (Figure 1b). The negative of light intensity (Figure 1c) is proportional to the fraction of absorbed light (AL) by the finger tissues and a second series was obtained as the intervals between AL maxima, which occur in the systolic phase of the beat (SSI). Furthermore, we calculated the first (Figure 1d) and second (Figure 1e) derivatives of AL. Since the derivative operator amplifies the high-frequency noise, the calculus was performed after low-pass filtering (fourth-order zero-phase Butterworth filter with 25 Hz cut-off frequency). Then we identified the intervals between maxima of the first (dP1) and second (dP2) derivative.

**Figure 2:**
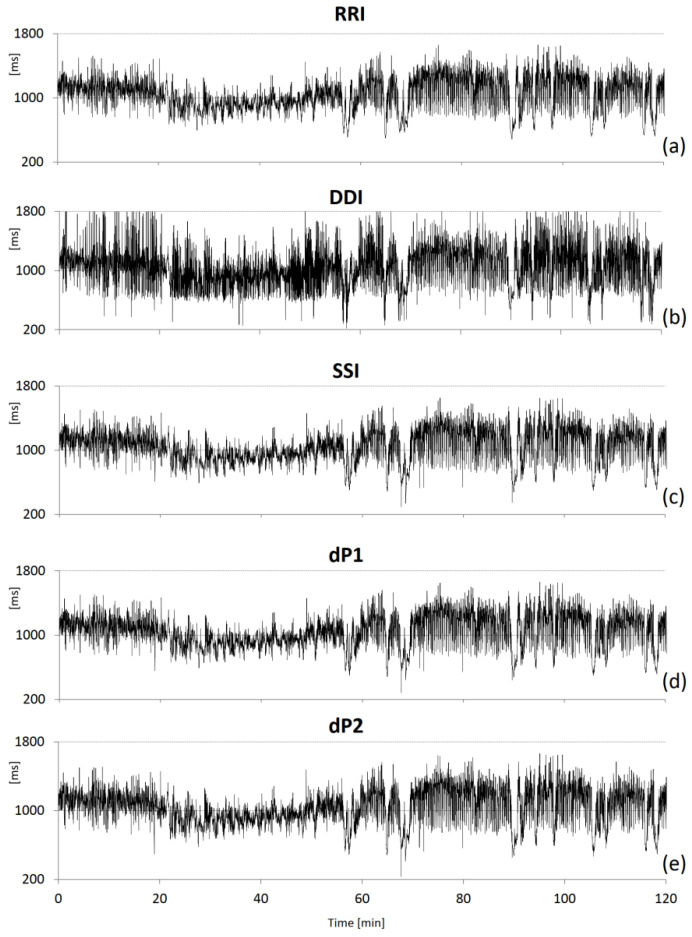
Example of IBI series during sleep at high altitude. (**a**) RRI from the ECG (reference); (**b**) DDI from the transmitted light (TL); (**c**) SSI from the absorbed light (AL = −TL); (**d**) dP1 from AL 1st derivative and (**e**) dP2 from AL 2nd derivative.

SSI, which is the peak-to-peak interval, is a commonly used PPG tachogram because it is defined similarly to RRI. DDI has been also proposed to measure the distance between consecutive feet of the pulse waves and is also called valley-to-valley or foot-to-foot interval [15,21,22]. The dP2 fiducial point is intended to identify the foot of the wave more precisely as the instant when the blood volume starts to rise after the heart contraction. The dP1 point is where the pulse wave upstroke is most rapid. Figure 2 shows an example of the beat-to-beat intervals derived from the ECG and PPG.

### 2.3. HRV Analysis

Beat-by-beat series were interpolated evenly at 5 Hz before spectral analysis. Power spectra were estimated by the Welch periodogram with 90% overlapped and linearly detrended Hann windows of 300 s length. The spectra were further broadband smoothed with a moving average whose order increased with the spectral frequency as in [23] and integrated over the very-low-frequency (VLF, between 0.003 and 0.04 Hz), low-frequency (LF, between 0.04 and 0.15 Hz), and high-frequency (HF, between 0.15 and 0.4 Hz) bands [4]. Discrepancies between PPG- and ECG-derived power spectra were quantified by the ratio between the spectrum of each PPG-derived tachogram (DDI, SSI, dP1, and dP2) and the RRI spectrum.

HRV complexity was quantified by self-similarity and entropy. The multiscale spectrum of self-similarity coefficients α(τ) was estimated as in [24] with the detrended fluctuation analysis code provided in [25]. The approach originally introduced in [24] consists in calculating the self-similarity scale coefficients as a continuous function of the temporal scale τ, in seconds, from the scale coefficients α_B_(*n*) evaluated on the “beat domain” as the local slope of the detrended fluctuation function log F(*n*) vs. log (*n*) [26]. This is done by mapping the scale units from the number of beats, *n*, to time τ, in seconds, with the transformation τ = *n* × μ_IBI_, with μ_IBI_ the mean inter-beat-interval of the series, in seconds. Short- and long-term coefficients, α_1_ and α_2_, respectively, were similarly calculated considering temporal scales and not scales defined on the beat domain. This was done averaging α(τ) over the scales 5 ≤ τ ≤ 12 s to calculate α_1_, and over 12 < τ ≤ 360 s to calculate α_2_. Discrepancies between PPG- and ECG-derived self-similarity spectra were quantified by the ratio between α(τ) of each PPG tachogram (DDI, SSI, dP1, and dP2) and α(τ) of RRI.

The sample entropy, SampEn, was estimated setting the tolerance *r* equal to 15% of the standard deviation of the series [27]. The multiscale entropy, i.e., SampEn as a function of the time scale τ in seconds, MSE(τ), was calculated for *m* = 1 and with a fixed tolerance *r* = 15% of the standard deviation [28]. Most of the literature on HRV evaluated SampEn with embedding dimension *m* = 2 following the original proposal in [27], but recently we showed that *m* = 1 provides the same type of information on the cardiovascular irregularity with more stable estimates [29]. Thus, this work shows the entropy estimates with *m* = 1 and interested readers will find the results for *m* = 2 in Appendix A.

We extracted three entropy indexes, MSE_HF_, MSE_LF_, and MSE_VLF_, averaging MSE(τ) over the scales corresponding to the HF (2.5 ≤ τ < 6.7 s), LF (6.7 ≤ τ < 25 s), and VLF (25 ≤ τ < 333 s) bands. Discrepancies between PPG- and ECG-derived multiscale entropies were quantified by the ratio between the MSE(τ) of each PPG tachogram (DDI, SSI, dP1, and dP2) and the MSE(τ) of RRI.

### 2.4. Statistics 

HRV indexes are tabulated as median (standard error of the median) unless otherwise stated. Comparisons between HRV indexes calculated for each PPG-derived tachogram and the reference RRI and comparisons between measures at sea level and high altitude were performed with the Wilcoxon matched-pairs rank test. Significance thresholds were set at 5% with a two-sided alternative hypothesis. Statistical analyses were performed with “R: A Language and Environment for Statistical Computing” software package (R Core Team, R Foundation for Statistical Computing, Vienna, Austria, 2021).

## 3. Results

### 3.1. Mt. Rosa Expedition

The recordings of oxygen saturation confirmed sleep-related breathing disorders with a broad range of severity, with the apnea-hypopnea index ranging between 0.1 and 10.2 events/hour in the subgroup taking acetazolamide and between 3.9 and 41.4 events/hour in the placebo subgroup. The duration of the sleeping period, selected for HRV analysis on the basis of stable triaxial accelerometer tracings, ranged between 60′ and 148′, with a mean (SD) equal to 101′ (27′).

After manual removal of premature beats and noisy segments, virtually the whole ECG signal was available for the analysis, the percentage of discarded ECG ranging from 0.0% to 0.2%. The percentage of discarded PPG was slightly but significantly (*p* < 0.01) greater, ranging between 0.1% and 5.5%. The median (SE median) RRI over the whole group was 813 (31) ms; the corresponding value for the PPG-derived tachograms (i.e., DDI, SSI, dP1, and dP2) was 815 (32) ms, the +2 ms difference with RRI being statistically significant (*p* < 0.01).

**Figure 3:**
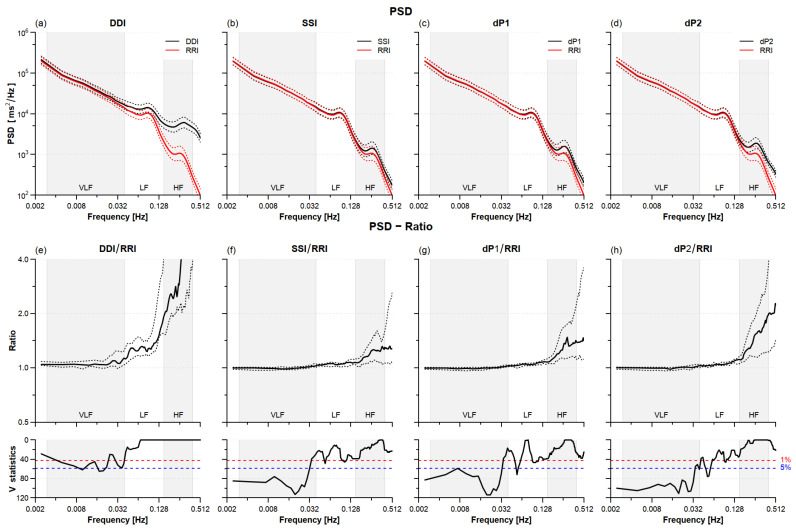
HRV power spectra density (PSD) sleeping at high altitude. Upper panels: geometric mean (solid line) ± geometric standard error (dotted line) for PSD calculated from DDI (**a**), SSI (**b**), dP1 (**c**), and dP2 (**d**) tachograms (black) and the reference RRI (red) over N = 21 participants. Lower panels: spectral ratios between DDI (**e**), SSI (**f**), dP1 (**g**), and dP2 (**h**) tachograms and the reference tachogram: median (solid line) with 1st and 3rd quartiles (dotted line) and V-statistics of Wilcoxon rank test; at frequencies where the V-value is above the 5% (or 1%) significance threshold (dashed horizontal line) the spectral ratio differs from 1 at the 5% (or 1%) statistical significance. Grey/white areas indicate the frequency bands for evaluating VLF, LF, and HF powers.

Figure 3 compares the power spectra of PPG tachograms with the reference RRI spectrum. The largest discrepancies regard DDI, whose spectrum is greater than the reference at all of the frequencies and particularly in the HF band, where it is more than twice the reference power. SSI, dP1, and dP2 power spectra are also greater than the reference at frequencies >0.03 Hz. SSI appears to provide the estimates closer to the reference with median power amplification in the HF band not greater than +32%. Table 1 shows that all of the PPG tachograms overestimated the LF and HF powers (DDI also overestimates the VLF power) and underestimate the LF/HF powers ratio.

**Figure 4:**
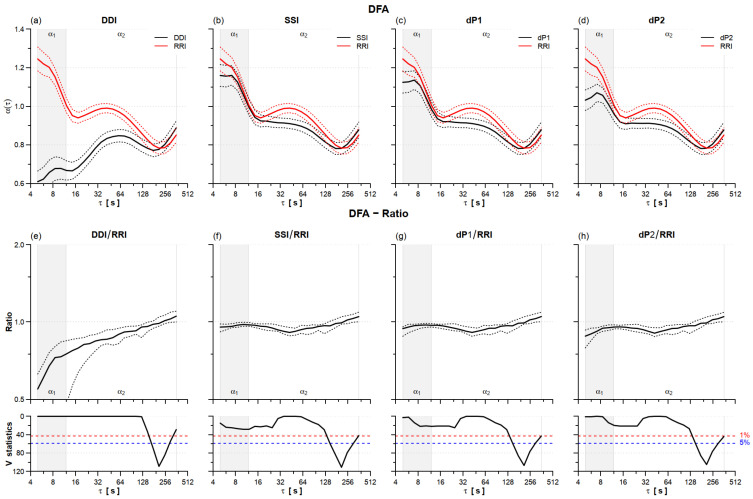
HRV detrended fluctuation analysis (DFA) sleeping at high altitude. Upper panels: mean (solid line) ± standard error (dotted line) for DFA spectra of DDI (**a**), SSI (**b**), dP1 (**c**), and dP2 (**d**) (black) and the reference RRI (red) on *n* = 21 participants. Lower panels: ratio between the DFA of DDI (**e**), SSI (**f**), dP1 (**g**), and dP2 (**h**) tachograms and the reference RRI: median (solid line) with 1st and 3rd quartiles (dotted line) and V-statistics of Wilcoxon rank test; at scales where the V-value is above the 5% (or 1%) significance threshold (dashed horizontal lines) the DFA ratio differs from 1 at the 5% (or 1%) statistical significance. Grey/white areas indicate the scales for evaluating α_1_ and α_2_ scale exponents.

All of the PPG tachograms, especially DDI, underestimated the self-similarity spectra at all scales shorter than 100 s (Figure 4). SSI, dP1, and dP2, substantially underestimated scales between 24 and 90 s, and dP2 also scales shorter than 10 s.

Table 1 reports significant underestimations for both the short-term and long-term scale exponents, α_1_ and α_2_, with the larger discrepancy for DDI.

**Figure 5:**
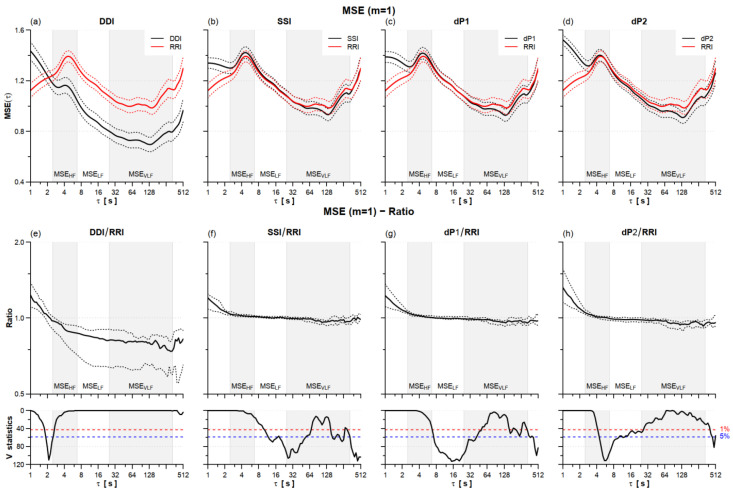
HRV multiscale entropy (MSE) for *m* = 1 sleeping at high altitude. Upper panels: mean (solid line) ± standard error (dotted line) for MSE of DDI, SSI, dP1, and dP2 (black) and the RRI reference (red) on *n* = 21 participants (panels (**a**–**d**)). Lower panels: MSE ratio between DDI (**e**), SSI (**f**), dP1 (**g**), and dP2 (**h**) tachograms and the RRI reference: median (solid line) with 1st and 3rd quartiles (dotted line) and V-statistics of the Wilcoxon’s rank test; at the scales where V is above the 5% (or 1%) significance threshold (dashed horizontal line) the ratio differs from 1 at the 5% (or 1%) statistical significance. Grey/white areas indicate the scales for evaluating MSE_HF_, MSE_LF_ and MSE_VLF_.

**Figure A1:**
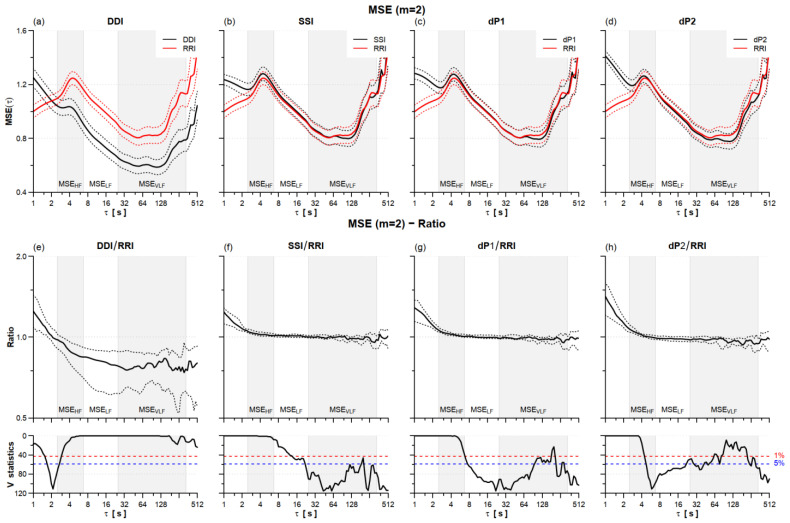
HRV multiscale entropy (MSE) for *m* = 2 sleeping at high altitude. Upper panels: mean (solid line) ± standard error (dotted line) for MSE of DDI, SSI, dP1, and dP2 (black) and the RRI reference (red) on N = 21 participants (panels (**a**–**d**)). Lower panels: MSE ratio between DDI (**e**), SSI (**f**), dP1 (**g**), and dP2 (**h**) tachograms and the RRI reference: median (solid line) with 1st and 3rd quartiles (dotted line) and V-statistics of the Wilcoxon’s rank test; at the scales where V is above the 5% (or 1%) significance threshold (dashed horizontal line) the ratio differs from 1 at the 5% (or 1%) statistical significance. Grey/white areas indicate the scales for evaluating MSE_HF_, MSE_LF_ and MSE_VLF_.

As for entropy, all of the PPG tachograms provided significantly greater SampEn values, the increase ranging between +22% for SSI and +35% for dP2 (Table 1 and Table A1). The greater differences in multiscale entropy (Figure 5 and Figure A1) regarded DDI that substantially overestimated scales shorter than 2 s and underestimated larger scales. SSI, dP1, and dP2 overestimated MSE at the shorter scales too but provided MSE profiles similar to the reference RRI at τ > 4 s, with just a slight but significant underestimation at τ around 100 s, more pronounced for dP2. Table 1 highlights a significant overestimation of MSE_HF_ and underestimation of MSE_VLF_ for SSI, dP1, and dP2.

### 3.2. Mt. Himalaya Expedition

The duration of the stable sleeping period selected for the analysis ranged between 60′ and 79′ at sea level, 65′ and 73′ at 6000 m. a.s.l., and between 62′ and 79′ at 6800 m a.s.l. After removal of premature beats and noisy segments, virtually the whole ECG segment was available for HRV analysis, the percentage of deleted ECG signal ranging between 0% and 0.2%, 0% and 0.1%, 0%, and 1.4% at Milan, Camp 1, and Camp 2, respectively. The percentage of deleted PPG signal was slightly greater: between 0.2% and 2.3%, 0.1% and 2.7%, and 0.1% and 7.3% at the three altitudes, respectively.

Over the group, the RRI was 944 (118) ms in Milan, 957 (86) ms in Camp 1, and 890 (24) ms in Camp 2, as mean (SD). DDI, SSI, dP1, and dP2 means were slightly and insignificantly greater, being 946 (119) ms in Milan, 961 (86) ms in Camp 1, and 895 (29) ms in Camp 2.

Table 2 compares HRV indexes at sea level and 6000 m a.s.l. The VLF, LF, and HF band powers by RRI increased significantly. The increase was also observed evaluating these powers by SSI, dP1, and dP2, while DDI did not detect a significant increase in the HF power. However, the SSI, dP1, and dP2 tachograms quantified a significant increase in the LF/HF powers ratio not revealed by RRI. Interestingly, α_1_ increased significantly for dP2 and showed a trend to increase (*p* < 0.15) for dP1 while its increase was far from the statistical threshold for the reference RRI (*p* = 0.69). Similarly, a decreasing trend was observed in SampEn estimated from SSI, dP1, or dP2 (*p* = 0.14), while the change of RRI SampEn was far from the statistical significance (*p* = 0.50). These trends characterize also the higher embedding dimension (*m* = 2, Table A2).

Table 3 describes the HRV changes in the four participants that reached Camp 2, reporting the ratio between measures at Camp 2 and sea level. It suggests that PPG quantifies increases in VLF and LF spectra similarly to RRI, while the increase in the HF power and decrease in LF/HF powers ratio is clearer in RRI than SSI, dP1, or dP2. Trends in complexity indexes quantified by SSI, dP1, and dP2, and by RRI, appear similar. The largest discrepancies with respect to the reference regard DDI, which quantifies relative changes substantially different from RRI not only for the HF power and LF/HF powers ratio but also for α_1_ and most entropy indexes, both for *m* = 1 (Table 3) and *m* = 2 (Table A3).

## 4. Discussion

The scientific literature on PPG as an ECG surrogate for HRV analysis suggests that discrepancies may depend on several factors: the PPG measurement site on the body, posture, activity level, the subjects’ general characteristics and possibly present pathological conditions, and the experimental setting. Our two experimental settings compared a wearable finger PPG system with the ECG in volunteers sleeping at high altitudes. On the one hand, they validated a wearable PPG device in an extreme environment; on the other hand, they allowed a better understanding of the usefulness of HRV analysis by PPG in high-altitude medicine and sleep disorders, the latter being risk factors for cardiovascular events with high incidence even in populations living at sea level. Our results testify to the capability of wearable PPG devices to provide sleep recordings of good quality even at very high altitudes but also point out discrepancies with the reference relevant for the correct interpretation of HRV changes. The rest of this section discusses the differences between HRV indexes from the PPG and the ECG, as resulting from the data of the Mt. Rosa expedition, and the consequences of such discrepancies in assessing changes occurring from sea level to high altitude, as resulting from the data of the Himalaya expedition.

### 4.1. Quality of PPG Recording during Sleep at High Altitude 

The finger PPG is very sensitive to movements artifacts but selecting the analysis periods based on stable tri-axial accelerometer tracings, only a small percentage of heartbeats were removed, even in the presence of severe sleep disorders (i.e., elevated apnea-hypopnea indexes). However, the percentage of removed beats was even lower for the ECG. For this reason, in some cases, the HRV measures from the ECG and PPG regarded a different number of beats. This explains why the mean cardiac intervals from the ECG and PPG were not identical. Even if the difference was a few milliseconds only (e.g., 813 vs. 815 ms for the Mt. Rosa group) without clinical relevance, the PPG cardiac interval was always greater. The removal of movement artifacts may have excluded heartbeats shorter than their neighboring beats because they were associated with the sympathetic activation induced by movements. This selection bias is expected to be more pronounced for PPG than ECG, ECG being less prone to this type of artifact. Only a small number of beats were removed, assuring that this possible bias did not affect our HRV measures relevantly. However, when parasomnias causing frequent body movements during sleep are present (such as restless leg syndrome or periodic limb movement disorder), the PPG might more importantly underestimate HRV indexes of cardiac sympathetic activity because of the removal of artifacts associated with sympathetic activations.

### 4.2. HRV Power Spectra

All of the PPG tachograms overestimated the HRV power spectrum at frequencies shorter than 0.03 Hz (Figure 3). The overestimation, more pronounced for the HF power, resulted in the lower LF/HF powers ratio (Table 2). A significantly greater HF power with a nonsignificant overestimation of the LF power was first described comparing the finger arterial pressure with the ECG [30] and explained as the consequence of changes in pulse wave velocity due to respiratory-induced oscillations in the arterial blood pressure. Our results could be similarly explained by modulations in the pulse wave velocity being particularly intense at the respiratory frequencies. Therefore, the interpretation of changes in the HF power and LF/HF powers ratio in terms of changes in cardiac autonomic control should be done with care when measures are obtained using both PPG and ECG signals.

### 4.3. HRV Self-Similarity 

The profile of self-similarity coefficients, α(τ), differed markedly from the reference RRI only for the DDI tachogram (Figure 4). However, also SSI, dP1, and dP2 had α_1_ and α_2_ exponents lower than the reference RRI. The short-term self-similarity coefficient, α_1_, is influenced by the HF and LF powers [31] and like the LF/HF powers ratio, reflects the sympathovagal balance, decreasing as the sympathetic tone decreases or the vagal tone increases [24,26]. Therefore, the same physiological mechanism causing the HF power overestimation and LF/HF powers ratio underestimation through modulations in pulse wave velocity could be responsible for the underestimation of α_1_.

### 4.4. HRV Entropy 

Only DDI provided estimates of multiscale entropy critically discrepant from the reference RRI at τ > 4 s (Figure 5 and Figure A1). However, all of the PPG series substantially overestimated SampEn and, as regards SSI, dP1, and dP2, also the MSE_HF_ index. SampEn has been directly linked to vagal heart rate modulations, decreasing after vagal blockade by atropine [32]. Therefore, the overestimation of SampEn by the PPG series appears coherent with the overestimation of the HF power and could be similarly associated with respiratory induced modulations in the pulse wave velocity.

### 4.5. Comparison among PPG Tachograms 

We found that the dynamics of DDI differ largely from the other PPG tachograms (SSI, dP1, and dP2), with important differences from the reference RRI. Other studies considered the minimum of the absorbed light waveform at the finger as the fiducial point, which indicates when the pulse wave starts rising. The intervals between pulse wave minima were also shown to correlate very well with RRI but in a rat animal model, where the peripheral pulse wave was the invasive femoral arterial pressure [33]. By contrast, in our volunteers sleeping at high altitudes, the absorbance minimum did not always coincide with the foot of the pulse wave and differed markedly from dP2, another fiducial point of the pulse wave foot (Figure 1). The reason is not a too low sampling rate (PPG and ECG are sampled at 200 Hz) or noise (the difference is evident also in the low-noise recording of Figure 1); more likely, the reason lies in hemodynamic effects in peripheral vessels, such as the superposition of direct and reflected waves and/or waveform distortions due to a nonlinear phase of the vascular transfer function. Even if the difference between DDI and dP2 might provide information on the vascular hemodynamic, our results highlight that DDI, at least in lying sleeping individuals, is a poor RRI surrogate for HRV analysis compared to SSI, dP1, and dP2.

Furthermore, our results suggest a more pronounced overestimation of the HF power and SampEn for dP1 than SSI, and for dP2 than dP1; and a more pronounced underestimation of LF/HF ratio and α_1_ for dP1 than SSI, and for dP2 than dP1. This could be due to residual high-frequency noise affecting the PPG signal. The first derivative, even if calculated after band-pass filtering, may have increased the effects of noise at the high frequencies, increasing the HF power, shifting the value of SampEn towards the higher value of uncorrelated noise, and shifting the value of α_1_ in the direction of the self-similarity exponent of white noise (i.e., 0.5). The second derivative may have amplified these effects. Accordingly, SSI should be preferred to dP1 and dP2 in the presence of noisy PPG recordings.

### 4.6. HRV Changes at High Altitudes 

Although the Mt. Rosa expedition highlighted substantial discrepancies between PPG and ECG in the spectral powers, nevertheless, when the professional mountaineers slept at 6000 m a.s.l., the SSI, dP1, and dP2 tachograms detected the same significant powers increases identified by RRI (Table 2). However, the PPG-derived tachograms also pointed out significant increases in the LF/HF powers and trends in α_1_ and SampEn absent in RRI. Therefore, the short-term dynamics of HRV might reflect different aspects of the physiological adaptation to high altitudes if the PPG is used as a surrogate for ECG. Furthermore, the data of the Himalaya expedition confirm that DDI is a poor surrogate for RRI compared to other PPG tachograms.

### 4.7. Limitations 

Since the discrepancies between PPG and ECG measures may be due to the distortion of the traveling pulse wave and to modulations of the pulse wave velocity, different results may characterize PPG signals measured at other sites than the finger, such as the more proximal earlobe [34] or distal toe, or reflectance PPG measures in the ear canal, on the wrist, or temple. Furthermore, since we hypothesize that the vascular characteristics may determine discrepancies between PPG-derived series and RRI, the results might differ in populations younger or older than the mid-age population of healthy volunteers considered in our work. Finally, the results we present in this work regarding the Himalaya expedition were obtained in a small number of participants. Although these data are useful to qualitatively describe how the use of PPG rather than ECG may critically influence the conclusions of sleep studies at high altitudes, the sample size is too small for solid statistical inferences and larger studies are needed to characterize HRV alterations during sleep at very high altitudes.

## 5. Conclusions

Wearable finger PPG systems can measure signals of sufficient quality and duration for evaluating the cardio-respiratory interactions even when sleeping in extreme high-altitude environments. Nevertheless, it should be considered that HRV measurements by PPG tachograms differ substantially from the reference RRI at the shorter scales, in the frequency domain being affected by the HF power and LF/HF powers ratio, and in the complexity domain being affected by the short-term self-similarity exponent and sample entropy. This is likely due to physiological and potentially informative modulations in the pulse wave transmission time. However, this systematic bias may make critical the interpretation of the faster HRV components, when HRV measurements from PPG and ECG signals are compared.

## Figures and Tables

**Table 1:** HRV indexes in *n* = 21 participants sleeping at high altitude: median (SE median) by type of tachogram.

	RRI	DDI	SSI	dP1	dP2
Power Spectral Analysis					
VLF (ms^2^)	1888 (478)	1915 (487) **	1860 (480)	1854 (481)	1855 (477)
LF (ms^2^)	986 (377)	1180 (523) **	1047 (391) **	1042 (381) **	1038 (392) **
HF (ms^2^)	383 (129)	1791 (375) **	424 (143) **	453 (142) **	498 (139) **
LF/HF	3.93 (0.57)	1.03 (0.32) **	2.96 (0.44) **	2.74 (0.38) **	2.51 (0.32) **
Detrended Fluctuation Analysis					
α_1_	1.158 (0.054)	0.700 (0.096) **	1.092 (0.043) **	1.076 (0.043) **	1.037 (0.035) **
α_2_	0.885 (0.029)	0.819 (0.028) **	0.848 (0.016) **	0.848 (0.018) **	0.846 (0.017) **
Entropy Analysis (m = 1)					
SampEn	1.135 (0.064)	1.427 (0.12) **	1.384 (0.04) **	1.415 (0.047) **	1.519 (0.044) **
MSE_HF_	1.342 (0.055)	1.110 (0.087) **	1.391 (0.052) **	1.386 (0.051) **	1.372 (0.056) **
MSE_LF_	1.205 (0.029)	0.916 (0.07) **	1.212 (0.033) *	1.211 (0.036)	1.192 (0.04) *
MSE_VLF_	1.114 (0.063)	0.775 (0.075) **	1.047 (0.072) **	1.042 (0.067) **	1.039 (0.06) **

The * and ** mark differences vs. RRI significant at *p* < 0.05 and *p* < 0.01, respectively.

**Table 2:** HRV indexes in *n* = 5 climbers sleeping in Milan (sea level) and Everest Camp 1 (6000 m a.s.l.) by type of tachogram.

	RRI			DDI			SSI			dP1			dP2		
	Milan	Camp 1	*p*	Milan	Camp 1	*p*	Milan	Camp 1	*p*	Milan	Camp 1	*p*	Milan	Camp 1	*p*
Power Spectra															
VLF (ms^2^)	2016 (1.13)	4827 (0.59) *	0.04	2137 (1.13)	5421 (0.59) *	0.04	2000 (1.1)	4598 (0.62) *	0.04	2164 (1.1)	5137 (0.61) *	0.04	2176 (1.1)	5128 (0.62) *	0.04
LF (ms^2^)	1433 (0.89)	4346 (0.61) *	0.04	1869 (0.77)	5008 (0.67) *	0.04	1553 (0.87)	4476 (0.6) *	0.04	1520 (0.86)	4391 (0.62) *	0.04	1522 (0.85)	4388 (0.62) *	0.04
HF (ms^2^)	251 (0.76)	765 (0.73) *	0.04	1627 (0.8)	3969 (0.9)	0.08	316 (0.75)	758 (0.81) *	0.04	365 (0.71)	856 (0.7) *	0.04	432 (0.68)	921 (0.69) *	0.04
LF/HF	5.70 (0.67)	5.68 (0.58)	0.69	1.15 (0.72)	1.26 (0.76)	0.89	4.91 (0.79)	5.91 (0.65) *	0.04	4.16 (0.68)	5.13 (0.55) *	0.04	3.52 (0.7)	4.76 (0.54) *	0.04
Detrended Fluctuation Analysis															
α_1_	1.24 (0.11)	1.31 (0.04)	0.69	0.62 (0.13)	0.76 (0.11)	0.50	1.20 (0.07)	1.26 (0.05)	0.22	1.19 (0.07)	1.26 (0.03)	0.14	1.14 (0.06)	1.24 (0.03) *	0.04
α_2_	0.85 (0.08)	0.91 (0.04)	0.89	0.73 (0.06)	0.86 (0.05)	0.22	0.79 (0.07)	0.90 (0.04)	0.35	0.79 (0.06)	0.90 (0.04)	0.35	0.79 (0.07)	0.90 (0.04)	0.35
Entropy Analysis (m = 1)															
SampEn	1.14 (0.19)	1.09 (0.07)	0.50	1.66 (0.16)	1.77 (0.10)	0.50	1.44 (0.14)	1.15 (0.07)	0.14	1.51 (0.18)	1.24 (0.06)	0.14	1.60 (0.16)	1.32 (0.05)	0.14
MSE_HF_	1.54 (0.13)	1.70 (0.05)	0.23	1.37 (0.15)	1.49 (0.08)	0.14	1.59 (0.10)	1.72 (0.03)	0.22	1.58 (0.10)	1.71 (0.03)	0.22	1.57 (0.03)	1.70 (0.03)	0.14
MSE_LF_	1.25 (0.14)	1.23 (0.06)	0.23	0.83 (0.25)	0.92 (0.10)	0.69	1.28 (0.13)	1.23 (0.06)	0.35	1.27 (0.13)	1.22 (0.06)	0.22	1.25 (0.06)	1.22 (0.06)	0.50
MSE_VLF_	1.14 (0.19)	1.16 (0.12)	0.50	0.89 (0.20)	0.95 (0.19)	0.22	1.13 (0.18)	1.19 (0.11)	0.35	1.12 (0.20)	1.20 (0.11)	0.35	1.13 (0.11)	1.19 (0.11)	0.35

Power Spectra as geometric mean (geometric SE), complexity indexes as median (SE median), *p* after Wilcoxon’s rank test (* highlights *p* values < 0.05).

**Table 3:** HRV indexes in *n* = 4 climbers: median [range] of the ratio between measures taken at Camp 2 and Milan.

	RRI	DDI	SSI	dP1	dP2
Power Spectra					
VLF	1.90 [0.89–8.12]	1.86 [1.00–7.10]	1.90 [0.91–8.05]	1.77 [0.90–7.08]	1.77 [0.84–7.08]
LF	2.89 [0.54–11.1]	2.90 [0.53–9.25]	2.82 [0.53–10.8]	2.85 [0.52–10.6]	2.88 [0.53–10.6]
HF	5.04 [1.03–7.36]	1.75 [0.64–11.9]	3.45 [1.00–6.94]	3.17 [0.87–6.52]	3.14 [0.75–5.92]
LF/HF	0.67 [0.47–1.50]	1.02 [0.28–6.17]	0.81 [0.53–1.55]	0.90 [0.60–1.63]	0.92 [0.70–1.78]
Detrended Fluctuation Analysis					
α_1_	0.95 [0.84–0.96]	1.02 [0.74–2.14]	0.96 [0.80–1.00]	0.98 [0.83–1.01]	0.99 [0.86–1.03]
α_2_	1.03 [0.82–1.19]	1.06 [0.95–1.17]	1.04 [0.87–1.19]	1.04 [0.87–1.20]	1.04 [0.88–1.20]
Entropy Analysis (m = 1)					
SampEn	0.89 [0.78–1.24]	1.09 [0.85–1.33]	0.86 [0.79–1.20]	0.84 [0.74–1.12]	0.90 [0.76–1.04]
MSE_HF_	1.07 [0.93–1.21]	1.09 [0.89–1.58]	1.06 [0.94–1.18]	1.07 [0.94–1.18]	1.07 [0.95–1.19]
MSE_LF_	0.99 [0.93–1.10]	1.06 [0.96–1.39]	0.99 [0.93–1.09]	0.99 [0.93–1.10]	1.00 [0.93–1.11]
MSE_VLF_	1.10 [0.73–1.34]	1.31 [1.21–1.39]	1.19 [0.75–1.43]	1.19 [0.75–1.46]	1.21 [0.76–1.47]

A ratio > 1 indicates a greater index at Camp 2 (6800 m a.s.l.) than at Milan (about sea level).

## Data Availability

The data that support the main findings of this study have been uploaded on the Zenodo repository at doi: 10.5281/zenodo.6415062 with access granted on justified request to researchers who meet the criteria for access to confidential data due to the restrictions requested for approval by the local ethical committee.

## Supplementary Materials

The following supporting information can be downloaded at: https://www.mdpi.com/article/10.3390/s22082891/s1, Table S1: General Characteristics of placebo and acetazolamide groups; Table S2: General Characteristics of placebo and telmisartan groups. (References [18,35] are cited in the Supplementary Material).

## Appendix A

Most studies on HRV entropy set the embedding dimension at *m* = 2 [27] but we showed that *m* = 1 provides similar information and more stable estimates [29]. Thus we selected *m* = 1 for estimating entropy in our study. This appendix presents entropy results for *m* = 2 showing that the discrepancies between the reference RRI and the PPG derived tachograms do not depend on the choice of *m*.

Figure A1 compares the multiscale entropies in the participants of the Mt. Rosa expedition displaying results similar to Figure 5.

Table A1 reports the entropy indexes for *m* = 2 in the participants of the Mt. Rosa expedition. Discrepancies between the reference RRI and each PPG tachogram are similar to those of Table 1.

**Table A1:** Entropy indexes for embedding dimension *m* = 2 in N = 21 participants sleeping at high altitude: median (SE median) by type of tachogram.

	RRI	DDI	SSI	dP1	dP2
SampEn	1.047 (0.074)	1.294 (0.097) **	1.295 (0.058) **	1.285 (0.052) **	1.419 (0.037) **
MSE_HF_	1.194 (0.061)	1.026 (0.065) **	1.262 (0.061) **	1.238 (0.058) **	1.194 (0.064) **
MSE_LF_	1.050 (0.054)	0.750 (0.084) **	1.082 (0.058) **	1.078 (0.056)	1.008 (0.053)
MSE_VLF_	1.056 (0.097)	0.639 (0.085) **	1.033 (0.079) *	1.017 (0.078) *	1.010 (0.086) **

The * and ** mark differences vs. RRI significant at *p* < 0.05 and *p* < 0.01 respectively.

Table A2 reports the changes from sea level to Camp 1 in entropy indexes calculated with *m* = 2 for the five mountaineers of the Himalaya expedition. Results are similar to those reported in Table 2.

**Table A2:** Entropy indexes for embedding dimension m = 2 in N = 5 climbers sleeping in Milan (sea level) and Everest Camp 1 (6000 m a.s.l.) by type of tachogram.

	Milan	Camp 1	*p*
SampEn			
RRI	1.07 (0.19)	0.96 (0.09)	0.22
DDI	1.52 (0.17)	1.49 (0.08)	0.69
SSI	1.40 (0.15)	1.07 (0.08)	0.08
dP1	1.46 (0.19)	1.12 (0.06)	0.14
dP2	1.53 (0.14)	1.23 (0.05)	0.14
MSE_HF_			
RRI	1.43 (0.18)	1.66 (0.05)	0.22
DDI	1.19 (0.17)	1.47 (0.07)	0.14
SSI	1.48 (0.15)	1.67 (0.04)	0.22
dP1	1.47 (0.15)	1.67 (0.04)	0.22
dP2	1.46 (0.04)	1.66 (0.04)	0.14
MSE_LF_			
RRI	1.13 (0.15)	1.17 (0.08)	0.22
DDI	0.71 (0.26)	0.91 (0.11)	0.89
SSI	1.15 (0.14)	1.15 (0.07)	0.22
dP1	1.15 (0.14)	1.15 (0.07)	0.22
dP2	1.13 (0.07)	1.15 (0.07)	0.22
MSE_VLF_			
RRI	1.08 (0.27)	1.13 (0.20)	0.35
DDI	0.66 (0.20)	0.77 (0.26)	0.22
SSI	1.07 (0.26)	1.16 (0.17)	0.35
dP1	1.06 (0.26)	1.16 (0.17)	0.35
dP2	1.07 (0.17)	1.16 (0.17)	0.35

*p* after Wilcoxon’s rank test.

Finally, Table A3 reports the ratios between measures at Camp 2 and sea level for entropy indexes estimated with *m* = 2 in the participants of the Himalaya expedition. As regards the differences between PPG-derived tachograms and the reference RRI, the results are similar to those of Table 3.

**Table A3:** Entropy indexes for embedding dimension *m* = 2 in *n* = 4 climbers: median [range] of the ratio between measures taken at Camp 2 and Milan.

	RRI	DDI	SSI	dP1	dP2
SampEn	0.86 [0.65–1.29]	1.02 [0.78–1.48]	0.82 [0.68–1.25]	0.81 [0.65–1.17]	0.88 [0.69–1.09]
MSE_HF_	1.09 [0.93–1.27]	1.13 [0.89–1.68]	1.08 [0.93–1.23]	1.10 [0.94–1.23]	1.11 [0.94–1.24]
MSE_LF_	1.01 [0.92–1.21]	1.13 [0.94–1.50]	1.00 [0.91–1.20]	1.01 [0.92–1.21]	1.02 [0.92–1.23]
MSE_VLF_	1.24 [0.73–1.64]	1.41 [1.25–1.65]	1.22 [0.78–1.68]	1.24 [0.79–1.72]	1.22 [0.79–1.75]

A ratio > 1 indicates a greater index at Camp 2 (6800 m a.s.l.) than at Milan (about sea level).
