# Supplementary material for: Heart Rate Variability from Wearable Photoplethysmography Systems: Implications in Sleep Studies at High Altitude
Source: Sensors (Basel). 2022 Apr 9;22(8):2891. doi: 10.3390/s22082891 (PMC9028181; doi:10.3390/s22082891)
Supplement: Supplementary file 1 [file sensors-22-02891-s001.zip › sensors-1639618-supplementary.pdf]

Mt. Rosa expedition, HIGHCARE-ALPS 2010
EudraCT No. 2010-019986-27

As described previously [18], forty-four healthy lowlanders (21 females) were included in a randomized, double-blind, parallel-group, placebo-controlled study. Participants were recruited among all the research teams involved in the previous Highcare expeditions, their families, friends, and acquaintances. After recruitment, the subjects were randomly assigned to receive either oral placebo or acetazolamide, 250 mg bid for 3 days at sea level in Milan, Italy (122 m a.s.l.) during the baseline tests and then throughout the entire duration of their stay at the high altitude laboratory located at Capanna Regina Margherita hut, Mt. Rosa (4'559 m a.s.l, barometric pressure 58.4 KPa, room temperature 19-20°C.), with pill intake being started the day before the ascent to high altitude. Only subjects without known cardiovascular disease, with no chronic cardiovascular therapy, no history of severe mountain sickness, no recent exposure to high altitude, and no contraindications to acetazolamide were included in the study. Women were requested to record their menstrual cycle in a dedicated diary and to avoid birth-control pills. The drugs were distributed as identical capsules containing 250mg of acetazolamide or placebo.
Ascent from Milan to the high-altitude laboratory was completed in less than 28 hours. Capanna Regina Margherita was reached from Alagna Valsesia (altitude 1'130 m a.s.l.) with subjects being first transported by cable car up to Punta Indren (3'200 m a.s.l.) and then hiking to Gnifetti hut (altitude 3'647 m a.s.l.). After an overnight stay at this altitude, subjects continued their hike up to Capanna Regina Margherita hut.
At high altitude, acute mountain sickness symptoms were evaluated every morning through the Lake Louise Score Questionnaire, where a score higher than 4 was taken as indicative of acute mountain sickness.
The sample size was calculated based on its primary aims, as discussed in [18].
Three subjects were excluded from the analysis: in the acetazolamide group one male did not ascend to high altitude for personal reasons and one female did not comply with the prescribed treatment; in the placebo group, one male did not reach the high-altitude laboratory. Thus, the study was performed on 20 subjects treated with acetazolamide and 21 subjects treated with placebo. Table S1 describes the general characteristics of the groups.

Table S1. General Characteristics of placebo and acetazolamide groups

	placebo group	acetazolamide group
**Sex (M/F)**	11/10	10/10
**Age, y/o**	37.3±10.1	34.8±7
**Weight, Kg**	67.0±12.9	63.7±11.7
**Height, cm**	172.2±9.6	172.7±8.8
**Body Mass Index, Kg/m^2**	22.5±2.7	21.3±2.7

Data as mean ±standard deviation

***Mt. Everest expedition*, HIGHCARE-HIMALAYA**
EudraCT No. 2008-000540-14

The extended protocol was published previously [35]. Briefly, fifty healthy lowlanders (including 10 alpinists with 1 female) aged <65 years (15 females) were included in a randomized, double-blind, parallel-group, placebo-controlled study examining the effects of telmisartan, a type-1 angiotensin II receptor blocker at high altitude. Participants were recruited among all the research teams involved in the previous Highcare expeditions, their families, friends, and acquaintances. The enrolled subjects were stratified by age and sex and randomized into blocks of four to receive either telmisartan 80 mg or placebo once daily in the morning. Exclusion criteria were a history of cardiovascular disease or arterial hypertension, a blood pressure ≥140/90 mmHg, any chronic cardiovascular therapy, exposure to high altitude in the preceding 8 months, a history of severe mountain sickness or angioedema, pregnancy, or lack of use of effective contraceptive methods in women in the fertile age range.
The drugs were distributed as identical capsules containing telmisartan 40 mg or placebo. The subjects started treatment by taking one morning capsule, moving to two-morning capsules after 2 weeks in the absence of adverse effects or marked abnormalities in some physiological parameters. After 8 weeks of treatment, participants traveled by air from Milan, Italy (120 m a.s.l.) to Kathmandu, Nepal (1’355 m a.s.l.) where they stayed for 3 days. They were then brought by air to Namche Bazaar (3’400 m a.s.l.), where they stayed for another 3 days. From there they hiked over 5 days to the Mt. Everest south base camp (5’400 m a.s.l.) where they remained for 12 days before returning to Milan in a 6 day-time, except for the alpinists who attempted to reach the summit of Mount Everest.
During the permanence at 3’400 and 5’400 m, subjects were not allowed to perform any further ascent and/or strenuous physical activity. Data collection was performed in tents under conditions of comfortable temperature at the Mt. Everest base camp, in a heated lodge at Namche Bazaar.
At high altitude, acute mountain sickness symptoms were evaluated every morning through the Lake Louise Score Questionnaire, where a score higher than 4 was taken as indicative of acute mountain sickness.
The sample size of this study was calculated based on its primary aims, as discussed in [35].
Three subjects (2 females) withdrew before the start of the study for personal reasons. Two subjects randomized in the telmisartan group (1 female) were excluded from the analysis because of scarce compliance to drug therapy. Thus, final data analysis was performed in 45 subjects (8 alpinists), 20 randomized to telmisartan (7 females), and 25 randomized to placebo (7 females).

Table S2. General Characteristics of placebo and telmisartan groups

	**placebo group**	**telmisartan group**
**Sex (M/F)**	18/7	13/7
**Age, y/o**	39.3±9.8	40.3±10.8
**Height, cm**	172.3±7.2	173.2±11.2
**Weight, kg**	65.8±9.7	71.1±15.4
**Body Mass Index, kg/m^2**	22.2±2.9	23.4±2.8

Data as mean ±standard deviation
